# Asymmetric Spread of SRBSDV between Rice and Corn Plants by the Vector *Sogatella furcifera* (Hemiptera: Delphacidae)

**DOI:** 10.1371/journal.pone.0165014

**Published:** 2016-10-19

**Authors:** Pei Li, Fei Li, Yongqiang Han, Lang Yang, Xiaolan Liao, Maolin Hou

**Affiliations:** 1 College of Plant Protection, Hunan Agricultural University, Changsha, 410128, China; 2 State Key Laboratory for Biology of Plant Diseases and Insect Pests, Institute of Plant Protection, Chinese Academy of Agricultural Sciences, Beijing, 100193, China; 3 Southern Regional Collaborative Innovation Center for Grain and Oil Crops in China, Changsha, 410128, China; University of Idaho, UNITED STATES

## Abstract

Plant viruses are mostly transmitted by sucking insects via their piercing behaviors, which may differ due to host plant species and their developmental stages. We characterized the transmission of a *fijivirus*, southern rice black-streaked dwarf virus (SRBSDV), by the planthopper vector *Sogatella furcifera* Horváth (Hemiptera: Delphacidae), between rice and corn plants of varying developmental stages. SRBSDV was transmitted from infected rice to uninfected corn plants as efficiently as its transmission between rice plants, while was acquired by *S*. *furcifera* nymphs at a much lower rate from infected corn plants than from infected rice plants. We also recorded a high mortality of *S*. *furcifera* nymphs on corn plants. It is evident that young stages of both the virus donor and recipient plants added to the transmission efficiency of SRBSDV from rice to corn plants. Feeding behaviors of the vector recorded by electrical penetration graph showed that phloem sap ingestion, the behavioral event that is linked with plant virus acquisition, was impaired on corn plants, which accounts for the high mortality of and low virus acquisition by *S*. *furcifera* nymphs on corn plants. Our results reveal an asymmetric spread of SRBSDV between its two host plants and the underlying behavioral mechanism, which is of significance for assessing SRBSDV transmission risks and field epidemiology, and for developing integrated management approaches for SRBSDV disease.

## Introduction

SRBSDV is a recently reported *Fijivirus* [1]. The white-backed planthopper (WBPH), *Sogatella furcifera* Horváth, is the only known vector and transmits SRBSDV in a persistent propagative manner [2]. In recent years, SRBSDV devastated rice crop in south China, Japan, Korea and Vietnam and corn crop in China and Vietnam [1,3–5]. The latent periods of SRBSDV in *S*. *furcifera* varies from 6 to 14 d and the minimum virus acquisition and inoculation access periods are 5 and 30 min, respectively [2]. Rice plants infected by SRBSDV present stunting and dark green leaves in the early stages and display small enations on stem and abnormal tiller formation on upper plant parts at the late stages [1]. The infected corn plants are stunted and dark green with very typical white waxy galls along veinlets on the underside of leaf blades [5]. Investigation shows that SRBSDV can infect a number of plant species, including gramineous plants, *Oryza sativa* L., *Echinochloa crusgalli* (L.), *Zea mays* L., *Paspalum distichum* L. and *Alopecurus aequali* Sobol., and cyperaceous plants *Juncellus serotinus* (Rottb.) C. B. Clarke and *Cyperus difformis* L. [6]. Adult *S*. *furcifera* could not transmit SRBSDV between gramineous plants and cyperaceous plants, but could transmit the virus among *O*. *sativa*, *E*. *crusgalli* and *Z*. *mays* [6]. However, despite of the economic importance of rice and corn crops for food security, SRBSDV transmission between these two crop species is still largely unclear.

Several factors or mechanisms may result in varying virus transmission between host plant species. Both plant- and animal-feeding insect vectors use volatile compounds derived from their hosts as key foraging cues [7–9], which can be quite different between the hosts and may be linked to varying virus transmission between the hosts [10]. And, virus transmission depends on a specific feeding behavior in the vector, which varies on different host plant species and may account for the varying transmission efficiency. Vector feeding behavior is usually revealed by observation of stylet penetration in the plant tissues [11,12] recorded using electrical penetration graph (EPG) [13].The western flower thrips *Frankliniella occidentalis* (Pergande) viruliferous with tomato spotted wilt virus (TSWV) showed more short ingestion probes on petunia *Petunia hybrida* Vilm and stramonium *Datura stramonium* L. than on tomato and pepper plants [14]. Further, different host plant species show differences in nutrition, amino acids, plant epidermis structure and vascular bundle [15]. These factors may further affect the transmission success of plant viruses via their influence on the vector and/or the transmitted virus.

In this study, we showed an asymmetric SRBSDV spread between rice and corn plants and stage-dependent transmission efficiency by the vector *S*. *furcifera*. Further, the feeding behaviors of WBPH on rice and corn plants, recorded using an EPG technique, were compared to test the hypothesis that differences in feeding behavior of the virus vector on different host plants underlie the observed asymmetric virus spread. These results add to our understanding of the underlying behavioral mechanisms responsible for the varying virus transmission and spread, and are of significance for assessment of SRBSDV field epidemiology and management of the virus disease.

## Material and Methods

### Insects and plants

Potted seedlings of a WBPH-susceptible rice variety (Taichung Native 1, TN1) and corn variety (Zhengdan 958) were cultured within 80-mesh insect-proof cages (50 by 50 by 50 cm) in a greenhouse (30 ± 5°C, 15 L: 9 D). The *S*. *furcifera* colony was maintained using caged rice seedlings in climate chambers (HP400GS, Wuhan Ruihua Equipment Co. Ltd, Wuhan, China) at 27 ± 1°C, 80 ± 5% relative humidity and a photoperiod of 15 L: 9 D.

Rice seedlings showing symptoms of SRBSDV were collected from paddy fields in May 2014 from Mangshi (24°25'53.02" N, 98°34'35.74" E), Yunnan Province, China or kindly provided by professor Guohui Zhou, South China Agricultural University. No specific permissions were required for the field collection of rice seedlings in the mentioned location because this collection did not involve endangered or protected or quarantine species. SRBSDV-positive seedlings, as determined by RT-PCR, were used to maintain a virus stock culture in cages in the greenhouse.

To obtain virus infected plants for the experiments, virus-free 15 d old rice or 3 d old corn seedlings were caged with viruliferous planthoppers at a density of two or three insects per plant for 3 d (rice) or 5 d (corn). Ten days thereafter, virus infection status of these rice and corn plants was confirmed individually by one-step RT-PCR. SRBSDV-positive plants were maintained in the greenhouse for subsequent use in assays.

### SRBSDV detection by RT-PCR

Virus infection status was detected by one-step RT-PCR [16]. Briefly, total RNA of each sample was extracted using the methods provided by the manufacturer of RNAiso Plus (Takara Biotechnology Co., Ltd, Dalian, China) [16]. The extracted RNA were amplified using primers (forward: 5’-cgcgtcatct caaactacag-3’, reverse: 5’- tttgtcagcatctaaagcgc-3’) [17] and PrimeScript One Step RNA RT-PCR Kit Ver.2 (Takara Biotechnology Co., Ltd, Dalian, China) according to the manufacturer’s instructions. The amplified fragment of the expected size (682 bp) of SRBSDV-S10 fragment (GenBank acc. No.EU523360.1) was confirmed by electrophoresis in 1% (w/v) agarose gels.

### SRBSDV transmission between rice and corn plants

In the tests for transmission efficiency of SRBSDV between rice and corn plants by *S*. *furcifera*, we employed rice plants at three development stages, i.e. three-leaf, tillering and booting stages, and corn plants also at three developmental stages, i.e. one-leaf, three-leaf and five-leaf stages. Transmission of SRBSDV from rice to corn plants or vice versa consists of acquisition of the virus by the vector from an infected plant species (donor plant) and, after a virus latent period, inoculation of the virus by the vector to an uninfected plant species (recipient plant).

In the test for SRBSDV transmission from rice to corn plants, nonviruliferous *S*. *furcifera* 4^th^ instar nymphs starved for 1 h were confined in a 10 ml tube encircling the stem of an infected rice seedling at three-leaf, tillering or booting stage for 48 h (acquisition access period, AAP) in the climate chambers. The insects were then transferred individually to three-leaf stage virus-free rice seedlings cultured in Kimura solution [16] in a glass tube (15 cm in length by 2 cm in diameter) in the climate chambers and maintained for 12 d (virus latent period). The Kimura solution was replenished when necessary. Then the planthoppers were individually trapped in a parafilm sachet (4.0 by 4.0 cm) onto a virus-free corn plant at one-leaf, three-leaf or five-leaf stage for 48 h (inoculation access period, IAP) in the chambers. Thereafter, the planthoppers were collected and individually detected by one-step RT-PCR for their virus infection status, and the corn plants exposed to viruliferous insects were transferred to insect-proof cages in the greenhouse and those exposed to nonviruliferous insects were discarded. The virus acquisition was tested to 312 planthoppers for each of the three rice stages. After a further 18 d, virus infection status of the corn plants exposed to viruliferous planthoppers was detected individually. The test for virus inoculation to corn plant was repeated for 86 to 93 times, each viruliferous planthopper obtained from a certain rice stage served as a replicate. The same procedures were used to test SRBSDV transmission from corn to rice plants.

### Feeding behaviors of WBPH adults and nymphs on rice and corn plants

In acquisition of SRBSDV from infected corn plants, the WBPH nymphs mostly died and the surviving nymphs acquired SRBSDV at extremely low rate from infected corn plants. To explain the unexpected results, we compared WPBH feeding amount and behaviors on rice and corn plants. The 4^th^ instar nymphs emerged as adults after AAP and the virus latent period in this study, and therefore both nymphs and adults were tested for their feeding amount and behaviors. Feeding amount was measured as honeydew excretion using a parafilm sachet method [18] with virus-free insects and plants. Two newly (< 24 h) emerged macropterous WBPH female adults or four 4^th^ instar nymphs starved for 1 h were placed in a parafilm sachet (4.5 by 4.5 cm) that was then fixed onto the stem of a tillering rice or three-leaf corn plant in the chambers (30 ± 1°C, 15 L: 9 D). The insects were removed from the sachet after 24 h and then the sachet was weighed immediately, and after removal of honeydew, weighed again using an electronic balance (Model XP205, Mettler Toledo, Shanghai, China). This allowed the net weight of honeydew excretion to be obtained. The tests were repeated for 24 times for each combination of WBPH stage (adult/nymph) and plant species (rice/corn).

The feeding behaviors of nonviruliferous 4^th^ instar nymphs and female adults (< 24 h) of WBPH on uninfected tillering rice and three-leaf corn plants were recorded. Although virus infection status is reported to influence planthopper’s feeding behaviors [19], our purpose here is to compare feeding behaviors between different plant species, therefore we used virus-free plants and insects. The recording was conducted using a Giga-8 direct current electrical penetration graph (DC-EPG) amplifier with a 10^9^-Ω input resistance in a Faraday cage (Wageningen University, Wageningen, The Netherlands) as reported by Lei et al. [19]. Briefly, a WBPH starved for 1.5 h (supplied with water only on filter paper in a flask) was immobilized using a micro-vacuum pump (QC-1S, Beijing Municipal Institute of Labour Protection, Beijing, China) by suctioning on its abdomen (nymphs were not exposed to this suction treatment) and then one end of a gold wire (18 μm diameter × 3–4 cm length) was attached to the dorsal thorax of the insect using a drop of water-soluble silver glue. The wired insect was then connected to the amplifier through a copper nail that was inserted into the EPG probe and placed on a rice or corn leaf sheath of a potted plant. A copper wire (2 mm diameter × 10 cm length) connected to the amplifier and vertically inserted into the pot soil was used as the plant electrode. The EPG signals were digitized with a converter (DI710-UL, Dataq, Akron, USA), and the gain of the amplifier was acquired and stored with the PROBE 3.4 software (Wageningen University, Wageningen, The Netherlands). The substrate voltage was adjusted so that the EPG signals fit into the +5 V to –5 V window provided by the PROBE software. Each insect was continuously recorded for 8 h. A different WBPH and plant combination was used for each recording. The EPG recording was conducted in a quiet room with ambient conditions of 25 ± 5°C and RH 20–30%. For each combination of WBPH stage (adult/nymph) and plant species (rice/corn), at least 12 valid repetitions were obtained.

**Fig 1:**
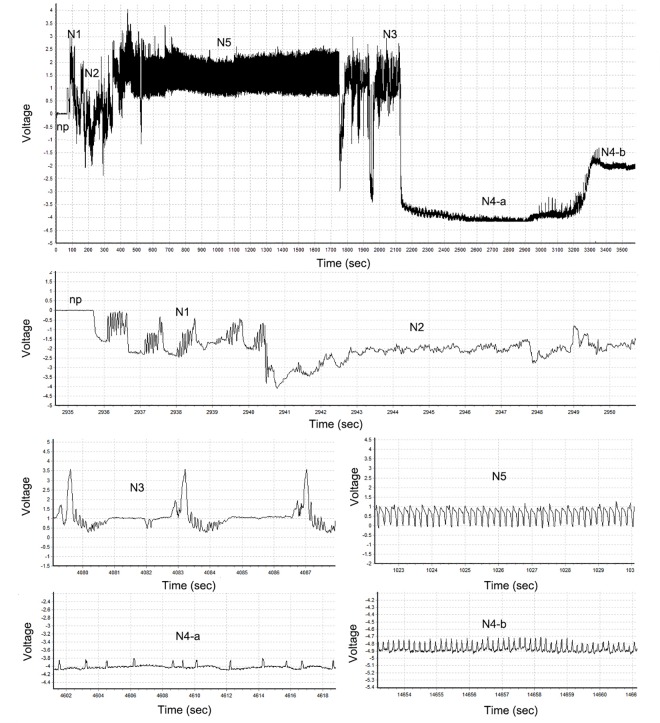
Typical EPG waveforms identified from *Sogatella furcifera* on rice and corn plants. np: non-penetration, N1: penetration initiation, N2: salivation and stylet movement, N3: extracellular stylet activity near the phloem region, N4-a: intracellular stylet activity in the phloem region without ingestion, N4-b: phloem sap ingestion, N5: stylets in the xylem tissue.

The recorded EPG waveforms were similar between the WBPH adults and nymphs. Seven distinctive waveforms were observed during probing by the WBPH in this study (Fig 1). These waveforms were interpreted with reference to Seo et al. [20], i.e., non-penetration (np), penetration initiation (N1), salivation and stylet movement (N2), extracellular stylet activity near the phloem region (N3), intracellular stylet activity in the phloem region without ingestion (N4-a) and phloem sap ingestion (N4-b), and stylets in the xylem tissue (N5). Four non-sequential variables were obtained from the EPG recordings [19, 21,22], i.e., number of waveform event per insect (NWEI), which is the sum of the number of events of a particular waveform in an insect; waveform duration per event (WDE, min), which is the sum of the durations of each event for a particular waveform divided by the number of events of that particular waveform; waveform duration per insect (WDI, min), which is the sum of the durations of each event of all the waveforms made by an insect; and proportion of individuals that produced sustained N4-b waveform (≥ 10 min). In addition, four sequential variables were also calculated from the EPG recordings [21,22], i.e., probing duration per probe (PDP, min), which is the sum of the probing duration of an insect divided by the number of probes made by the insect; total duration of N4-a followed by N4-b (min); duration from the beginning of a probe to the 1^st^ sustained N4-b (min); and total duration of N4-a followed by sustained N4-b (min).

### Statistical analysis

Virus acquisition and inoculation (S1 File) were compared between plant stages by a binomial logistic regression analysis (SPSS, version 19.0, IBM SPSS Statistics Inc., Chicago). In analysis of virus acquisition, insect infection status was used as a binomial response variable and donor plant stage, as an explanatory variable. In analysis of virus inoculation, infection status of recipient plant was used as a binomial response variable and donor plant stage and recipient plant stage, as explanatory variables. The variables of EPG recordings and feeding amount were compared between rice and corn plants by Student T-test for Gaussian distribution variables and Mann Whitney U-test for non-Gaussian distribution variables, and the proportion of individuals that produced sustained N4-b waveform was analyzed using the Chi-square 2 × 2 goodness of fit test or a Fisher exact test when the expected values were lower than 5.

## Results

### SRBSDV transmission from rice to corn

**Fig 2:**
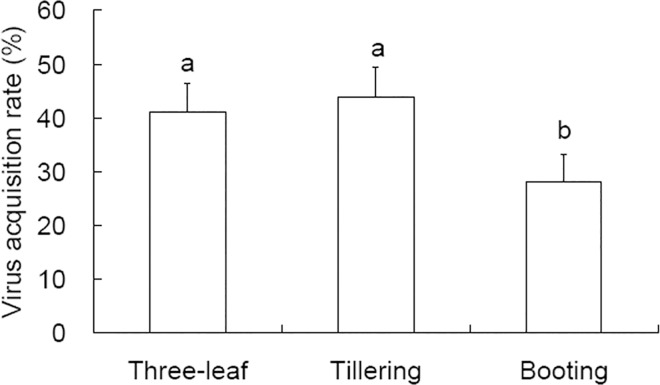
SRBSDV acquisition rate by *Sogatella furcifera* 4^th^ instars from infected rice plants at different developmental stages. The test was administered to 312 insects for each of the three rice stages. The data are expressed as means + 95% CI. Different letters over the bars indicate significant difference between treatments by binomial logistic regression.

After a 48 h AAP on infected rice plants and a 12 d virus latent period, the nonviruliferous *S*. *furcifera* nymphs were determined to have acquired SRBSDV at significantly different rates (28.2%-43.9%) from infected rice plants at three-leaf, tillering and booting stages (Fig 2). Virus acquisition was lower from rice plants at booting stage (88/312) than at three-leaf stage (132/312; OR = 1.867; df = 1; Wald = 13.452; *P* < 0.001) and tillering stage (137/312; OR = 1.993; df = 1; Wald = 16.484; *P* < 0.001) (Fig 2). Thus rice plants at three-leaf stage and tillering stage are more likely to serve as virus donor plants in paddy fields.

**Fig 3:**
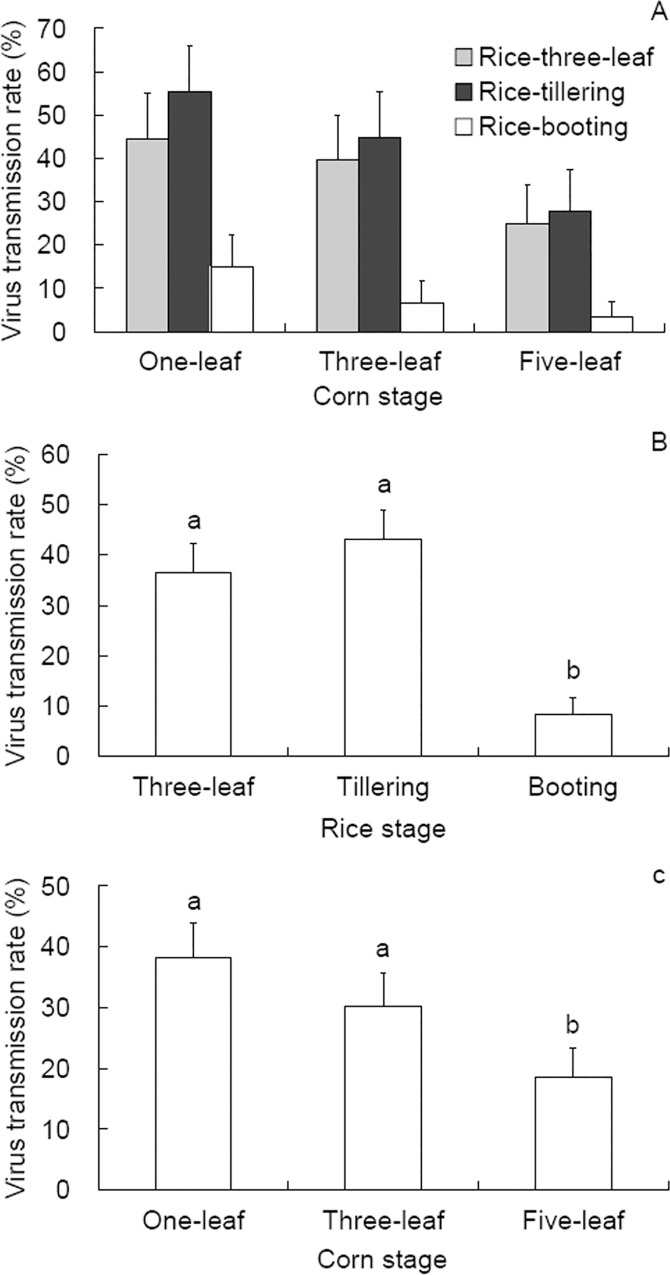
SRBSDV inoculation rate on corn plants by viruliferous *Sogatella furcifera* that acquired the virus from rice plants. (A) all combinations of rice and corn stages, (B) rice stage main effects, and (C) corn stage main effects. The test was repeated for 86 to 93 insects for each of the nine combinations of rice and corn stages. The data are expressed as means + 95% CI. Different letters over the bars in panel B and C indicate significant difference between treatments by binomial logistic regression.

After acquiring SRBSDV from infected rice plants, *S*. *furcifera* inoculated the virus to corn plants at rates between 3.3%-55.6% (Fig 3). A significant effect of rice stage (*P* < 0.001) and corn stage (*P* < 0.001) on SRBSDV inoculation success was detected, and a significant interaction between them was also observed (*P* < 0.001; Fig 3A). Specifically, the planthoppers that acquired SRBSDV from rice plants at three-leaf and tillering stages inoculated the virus to corn plants of different stages at higher rates (101/277; OR = 6.682; df = 1; Wald = 55.699; *P* < 0.001 for AAP on three-leaf and 114/265; OR = 8.833; df = 1; Wald = 73.273; *P* < 0.001 for AAP on tillering rice stage) than those insects that acquired the virus from booting stage rice plants (23/276; Fig 3B). These results indicate that young stage of virus donor plant contributes to successful virus inoculation to recipient plant by the vector. With respect to the effects of corn stage on SRBSDV transmission, the planthoppers inoculated the virus at higher rates to one-leaf corn plants (105/275; OR = 3.046; df = 1; Wald = 27.594; *P* < 0.001) and three-leaf corn plants (83/274; OR = 2.023; df = 1; Wald = 10.759; *P* = 0.002) than to five-leaf corn plants (50/269) (Fig 3C). Therefore young corn plants in the field are at high risks of being inoculated with SRBSDV.

### SRBSDV transmission from corn to rice

When the nonviruliferous *S*. *furcifera* 4^th^ instars were confined for 48 h to infected corn plants for virus acquisition, they died mostly at 90%. After a 12 d virus latent period, the surviving nymphs were detected to be viruliferous at less than 2.3%, which indicates that *S*. *furcifera* 4^th^ instars can hardly acquire SRBSDV from infected corn plants. Due to only a few viruliferous WBPH resulted from AAP on infected corn plants, inoculation of the virus by WBPH to rice plants was not measured.

### Feeding amount

**Fig 4:**
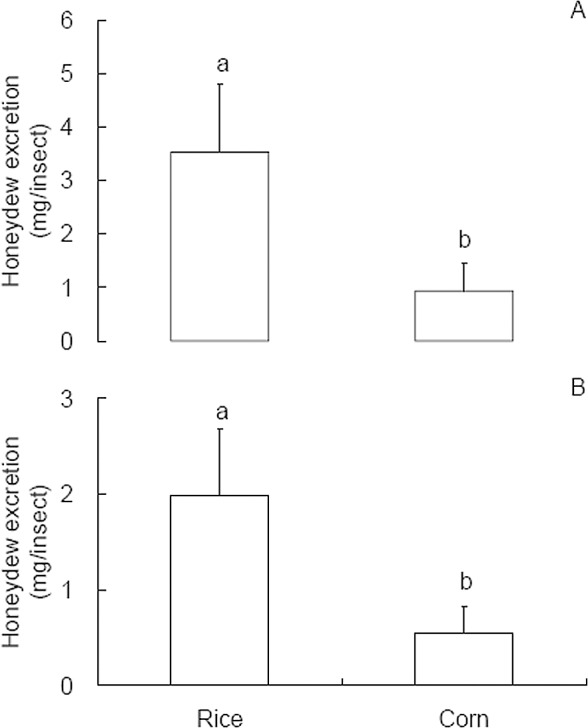
**Honeydew production of *Sogatella furcifera* adults (A) and nymphs (B) feeding for 24 h on rice and corn plants.** Values are means + 95% CI from 24 replicates. Different letters over the bars indicate significant difference (*P* < 0.05) by Student t-test.

Both WBPH adults and nymphs feeding on rice plants produced 3–4 times more honeydew than those feeding on corn plants (Fig 4; *t* ≥ 3.889, *P* ≤ 0.001), indicating that rice is a more suitable host plant than corn for the herbivore.

### Feeding behaviors of WBPH recorded by EPG

Values of non-sequential variables of WBPH feeding behaviors recorded by EPG were summarized in Table 1. Both WBPH nymphs and adults showed greater numbers of waveform events per insect of extracellular stylet activity near the phloem region (N3) (NWEI; nymphs: *t* = 5.00, *P* < 0.001; adults: *U* = 145.00, *P* < 0.001) and intracellular stylet activity in the phloem region (N4-a) (NWEI; *U* = 143.00, *P* < 0.001) on corn plants than on rice plants. With respect to waveform duration, both the adults and nymphs were characterized by a shorter N4-b waveform duration per insect (WDI; *U* ≥ 13.00, *P* ≤ 0.003) and waveform duration per event (WDE; U ≥ 10.00, *P* < 0.001) and N3 WDE (*U* ≥ 23.50, *P* ≤ 0.004) on corn than on rice plants, similar pattern were found in N4-a WDI (*t* = 3.042, *P* = 0.006) and WDE (*U* = 39.50, *P* = 0.036) of the adults and WDE (*U* = 13.00, *P* < 0.001) of the nymphs. In addition, the nymphs spent shorter time in non-penetration (np; WDI and WDE; *U* ≥ 31.00, *P* ≤ 0.007) while longer time in salivation and stylet movement (N2; WDI: *t* = 2.99, *P* = 0.006; WDE: *U* = 151.50, *P* = 0.003) and in xylem tissue (N5; WDI and WDE; *t* ≥ 2.293, *P* ≤ 0.031) on corn plants than on rice plants. The proportion of nymphs that produced sustained N4-b waveform was significantly lower on corn plants (6/13) than on rice plants (13/14) (Chi-Square test, *P* = 0.026).

**Table 1:** Non-sequential EPG variable values of the probing behavior of *Sogatella furcifera* nymphs and adults feeding on rice and corn plants during an eight-hour recording.

Waveforms	Plant	WBPH nymph			WBPH adult		
		NWEI	WDI	WDE	NWEI	WDI	WDE
np	Rice	15.9±1.5 a	50.9±7.7 a	3.4±0.5 a	12.0±1.5 a	39.3±9.8 a	3.0±0.5 a
	Corn	13.5±0.9 a	23.6±3.7 b	1.8±0.4 b	13.5±2.7 a	23.7±3.4 a	2.5±0.6 a
N1	Rice	6.7±0.9 a	2.0±0.3 a	0.3±0.0 a	6.5±1.0 a	4.2±2.3 a	0.6±0.3 a
	Corn	6.0±1.0 a	1.9±0.7 a	0.5±0.2 a	9.0±2.1 a	1.9±0.7 a	0.2±0.0 b
N2	Rice	23.4±2.1 a	49.0±6.9 b	2.1±0.2 b	19.4±1.8 a	58.6±6.7 a	3.1±0.2 a
	Corn	25.3±1.7 a	79.1±7.3 a	3.1±0.2 a	26.5±5.2 a	85.7±14.9 a	3.4±0.4 a
N3	Rice	13.8±2.2 b	63.0±9.7 a	5.2±0.9 a	10.5±1.0 b	53.4±5.8 a	5.4±0.7 a
	Corn	32.5±3.1 a	83.0±7.4 a	2.8±0.4 b	31.8±5.1 a	82.1±10.5 a	3.1±0.6 b
N4-a	Rice	7.6±1.9 b	63.8±10.7 a	10.4±1.2 a	5.7±0.7 b	48.7±1.8 b	10.5±1.9 a
	Corn	25.4±2.7 a	77.9±8.8 a	3.5±0.5 b	23.2±4.3 a	78.5±6.6 a	5.2±1.2 b
N4-b	Rice	2.6±0.4 a	152.0±18.4 a	82.7±18.7 a	2.7±0.3 a	173.8±26.0 a	78.0±19.2 a
	Corn	5.3±1.3 a	32.2±8.3 b	4.9±1.5 b	4.0±0.7 a	66.6±22.0 b	16.6±5.4 b
N5	Rice	6.1±0.7 a	99.3±14.8 b	17.8±2.5 b	5.1±0.6 a	100.5±14.6 a	21.1±2.8 a
	Corn	7.2±0.6 a	182.2±17.1 a	26.4±2.8 a	6.8±1.0 a	100.9±22.6 a	25.0±4.4 a

Values are means ± SE from 12–14 replicates. Different letters following the values of a variable for a certain waveform in WBPH nymphs or adults indicate significant difference (*P* < 0.05) between the two host plants by Student t-test (underlined) or Mann Whitney U-test (not underlined).

Table 2 shows sequential EPG variables that describe the sequence of events related to each other during the eight hours of recording. Both WBPH nymphs and adults showed longer total duration of N4-a followed by N4-b on rice plants than on corn plants (*U* ≥ 40.00, *P* ≤ 0.039). Similar pattern was also found in total duration of N4-a followed by sustained N4-b on rice plants than on corn plants (*U* ≥ 20.50, *P* ≤ 0.004). And the duration from the beginning of a probe to the 1^st^ sustained N4-b in WBPH nymphs is longer on corn plants than on rice plants (*U* = 49.50, *P* = 0.041).

**Table 2:** Sequential EPG variable values of the probing behavior of *Sogatella furcifera* nymphs and adults feeding on rice and corn plants during an eight-hour recording.

Variables	WBPH nymph		WBPH adult	
	Rice	Corn	Rice	Corn
Probing duration per probe (min)	31.1±3.3 a	35.3±2.2 a	45.7±7.3 a	55.7±12.2 a
Total duration (min) of N4-a followed by N4-b	45.0±8.9 a	21.3±6.7 b	40.5±7.4 a	25.7±3.3 b
Duration (min) from the beginning of a probe to the 1^st^ sustained N4-b	54.5±6.8 b	60.4±16.4 a	45.0±4.9 a	68.1±21.3 a
Total duration (min) of N4-a followed by sustained N4-b (≥ 10 min)	40.4±7.7 a	14.0±9.0 b	36.4±5.8 a	18.1±4.1 b

Values are means ± SE from 12–14 replicates. Different letters following the values of a variable in WBPH nymphs or adults indicate significant difference (*P* < 0.05) between the two host plants by Student t-test (underlined) or Mann Whitney U-test (not underlined).

## Discussion

In the present study, we recorded differential SRBSDV transmission between rice and corn plants. SRBSDV genes show similar expression patterns in distinct hosts [23], indicating that the varying SRBSDV transmission efficiency between host species originates from the vector’s host-associated behaviors. During AAP, *S*. *furcifera* 4^th^ instars acquired SRBSDV at a high rate from infected rice plants while at a low rate from infected corn plants. In a test using *S*. *furcifera* adults to acquire SRBSDV from 4–5 leaf stage corn plants, the virus acquisition rate was only 3.3% [2]. These results were confirmed by the EPG data (Table 1). Both nymphs and adults of *S*. *furcifera* showed shorter duration (both WDI and WDE) of sap ingestion (N4-b) on corn plants than on rice plants. Virus acquisition is linked to sap ingestion [24–27]. Therefore, it is clear that short sap ingestion has resulted in the low virus acquisition rate in the vector on the corn plants. Our results here respond to a previous report that successful acquisition of SRBSDV by *S*. *furcifera* on rice plants depends on extended phloem sap ingestion [19]. Similar results were also reported in the acquisition of barley yellow dwarf virus (BYDV) by *Rhopalosiphum padi*, where non-viruliferous *R*. *padi* acquired BYDV at a higher rate when the aphids generated more E2 waveforms (phloem sap ingestion) on BYDV-infected wheat plants [24]. SRBSDV titer varies between plants of the same species, between plant species [23], and between insects (unpublished data). In a non-persistent virus sweet potato feathery mottle virus (SPFMV), positive correlation exists between virus titer and transmission [28], while in a semi-persistent virus cucurbit yellow stunting disorder virus (CYSDV), no such correlation is observed [29], And to our knowledge, there are no reports about the role of virus titer in vector’s virus acquisition in persistent virus like SRBSDV. Therefore it seems that the low SRBSDV acquisition on corn plants cannot be attributed to possible low virus titer in corn plants. However, the low SRBSDV acquisition rate on corn plants found in this study and reported by Pu [2] contrasts to that by Li et al. [6], who reported a much higher SRBSDV transmission rate from infected corn plants to healthy rice plants and thus a presumable high virus acquisition rate from corn plants. The difference lies in that five insects were released per potted donor (corn) plants in a netting greenhouse and the insects were left to feed and move freely between the potted corn and encircling recipient (rice) plants in Li et al.’s study [6], while in our experiment and Pu’s report, the insects were individually confined with a corn plant in a parafilm sachet, the latter situation is a more realistic reflection of solid plantation in the field.

During IAP on corn plants, *S*. *furcifera* that acquired SRBSDV from rice plants were able to inoculate the virus, with inoculation rate comparable with reported inoculation rates on rice plants [2] and corresponds to the high occurrence of SRBSDV on corn plants in the field [5,30]. Persistent propagative viruses like SRBSDV are inoculated from viruliferous vector to plant via salivation [31,32]. In a previous study, we reported successful inoculation of SRBSDV to rice plants by *S*. *furcifera* relied on extended salivation and frequent stylet activities before phloem sap ingestion [19]. In the BYDV-*R*. *padi*-barley pathosystem, viruliferous *R*. *padi* that transmitted the virus to barley plants were characterized by longer salivation duration than those that failed to [24]. And transmission of TYLCV by viruliferous whiteflies to tomato plants was higher in insects that salivated into the phloem sap than those did not [26]. In this study, *S*. *furcifera* showed more stylet activities near and in the phloem region (N3 and N4-a *NWEI*; for both adults and nymphs) and longer salivation (N2) duration in the nymphs (both WDI and WDE) on corn plants than on rice plants (Table 1), while no differences were detected in probing attempts (N1 *NWEI*; for both adults and nymphs), salivation (N2) number (*NWEI*; for both adults and nymphs) and duration (WDI; for adults) between the two host plant species (Table 1). N1 and N2 are prerequisites for virus inoculation. Therefore, these results of probing behaviors of *S*. *furcifera* nymphs substantiate the comparable SRBSDV inoculation rates on corn plants with those on rice plants.

In virus acquisition from infected corn plants, *S*. *furcifera* nymphs died mostly. Pu et al. [2] recorded a 100% mortality of *S*. *furcifera* nymphs feeding for 48 h on diseased corn plants at 5–6 leaf stage, while *S*. *furcifera* adults all survived. Our EPG recordings indicated that *S*. *furcifera* (both nymphs and adults) spent longer time in stylet pathway (N1+N2+N3) and intracellular stylet activity in the phloem region (N4a) (Table 2) and shorter time in phloem ingestion (Table 1), thus produced less honeydew (Fig 4), on corn plants than on rice plants. Although corn is recorded as a host plant for *S*. *furcifera* [1,33,34], our results may suggest that corn is not a suitable host or is only an occasional host [35] for *S*. *furcifera*. Calatayud et al. [35] reported that the intracellular punctures were short and phloem finding and subsequent ingestion were markedly delayed on occasional hosts. Similar results were also reported for rice plants resistant to *Nilaparvata lugens*, where the insects spent less time ingesting phloem on plants carrying the resistance genes *Bph14* and *Bph15* than on susceptible plants [36]. These results indicate that the high mortality of *S*. *furcifera* nymphs on corn plants also resulted from impaired phloem sap ingestion, like the low virus acquisition rate on corn plants.

In the tests, we used rice plants to rear the insects and to maintain SRBSDV stock culture originating from rice plants. This may result in possible bias due to acclimatization on rice plants. However, SRBSDV gene expression patterns in distinct hosts are similar [23], therefore the possiblity of bias in virus transmission due to acclimatization of SRBSDV on rice plants can be excluded. WBPH is never recorded at high population size on corn plants in the field and the insects can only survive at a very low rate on corn plants. If we had reared the insects using corn plants, we could hardly have obtained sufficient test insects. It needs further tests for possible bias in SRBSDV transmission efficiency due to vector’s rearing plant species.

An effect of plant developmental stages on SRBSDV transmission by *S*. *furcifera* nymphs was detected in this study. During AAP from rice plants, virus acquisition rates were higher from younger plants than from older plants (Fig 2). Similar result was reported by Liu et al. [37] for the same plant-vector-virus system. Significant effects of both rice and corn developmental stage on SRBSDV inoculation to corn plants was detected (Fig 3), the younger the donor and the recipient plants, the higher the inoculation rates. When SRBSDV was inoculated into rice plants, younger plants were also inoculated at higher rates [38,39]. In another pathosystem, MRCV can be inoculated to corn plants only during their early developmental stages [40]. The host plant stage-dependent virus transmission efficiency may result from differential probing behaviors of the vector on plants of different ages, which deserves further tests.

Overall, our findings demonstrate an asymmetric spread of SRBSDV between rice and corn plants transmitted by *S*. *furcifera* nymphs, i.e., SRBSDV can be transmitted from infected rice to uninfected corn plants as efficiently as its transmission between rice plants, but can be acquired at a much lower rate from infected corn plants than from infected rice plants. Besides, we recorded a high mortality of *S*. *furcifera* nymphs on corn plants. The high mortality and low virus acquisition on corn plants can be of great significance in SRBSDV transmission and spread, because corn plants may act as a ‘sink’ for both *S*. *furcifera* and SRBSDV. We further show that impaired phloem sap ingestion by *S*. *furcifera* nymphs underlies their high mortality and low virus acquisition on corn plants. Additionally, it is evident that young stages of both the virus donor and recipient plants add to the transmission efficiency of SRBSDV from rice to corn plants. Given that SRBSDV is a threatening plant virus for rice production in Asia [41], these findings can provide fundamental information for assessing SRBSDV transmission risks and field epidemiology, and for developing integrated management approaches for SRBSDV disease.

## Supporting Information

S1 File Data obtained in the observation. (XLS)
